# GPs´ Personal Spirituality, Their Attitude and Spiritual Competence: A Cross-Sectional Study in German General Practices

**DOI:** 10.1007/s10943-022-01536-2

**Published:** 2022-04-27

**Authors:** Ruth Mächler, Cornelia Straßner, Noemi Sturm, Johannes Krisam, Regina Stolz, Friederike Schalhorn, Jan Valentini, Eckhard Frick

**Affiliations:** 1 Professorship of Spiritual Care and Psychosomatic Health, Department of Psychosomatic Medicine and Psychotherapy, University Hospital rechts der Isar, Technical University of Munich, Langerstr. 3, 81675 Munich, Germany; 2 Department of General Practice and Health Services Research, University Hospital Heidelberg, Im Neuenheimer Feld 130.3, 69120 Heidelberg, Germany; 3 Department for Medical Biometry, Institute of Medical Biometry and Informatics, University Hospital Heidelberg, Im Neuenheimer Feld 130.3, 69120 Heidelberg, Germany; 4 Institute of General Practice and Interprofessional Care, University Hospital Tübingen, Osianderstr. 5, 72076 Tübingen, Germany

**Keywords:** Spirituality, Competency-based education, General practitioners, General practice, Health services for the aged

## Abstract

To understand if GPs’ spiritual competence, their personal spirituality and attitude towards enquiring about spirituality in practice interrelate, we conducted a cross-sectional survey of 30 German GPs regarding issues of SC. We found correlations between GPs’ personal spirituality, their spiritual competence and their attitudes towards SC. The ability to perceive spiritual needs of patients was the competence most strongly related to GPs’ attitude towards SC. The competence with the strongest correlation to personal spirituality was Self-awareness and Proactive opening. No correlation was found between affiliation to a spiritual community and GPs’ attitude towards SC. The results show that GPs’ personal spirituality and spiritual competence are indeed related to addressing spirituality with their patients. To foster SC, training programmes should raise awareness for one’s personal spirituality and encourage one to reflect on spiritual competence.

## Introduction

Most General Practitioners (GPs) acknowledge that spirituality should be an integral part of primary care (Appleby et al., 2019; Assing Hvidt et al., 2016; Monroe et al., 2003). At the same time, GPs report lacking competencies to fulfil this task. The latter include discerning, addressing and meeting patients’ spiritual needs, being knowledgeable about the meaning of spirituality and religion, as well as being empathetic and appreciating patients’ thoughts and feelings (Frick et al., 2019; Hodge, 2007). Many patients and healthcare providers have voiced their desire to increase doctors’ spiritual competence (Balboni et al., 2007; Bar-Sela et al., 2019; Best et al., 2015; Büssing et al., 2009; Ellis et al., 1999; Frick et al., 2006).

In the late 1990s, the *Association of American Medical Colleges* encouraged physicians to talk about spirituality with their patients. However, the willingness to implement SC seems to depend on the physicians’ religious/spiritual (r/s) orientation. Religious physicians are more likely to discuss r/s issues with their patients (Curlin et al., 2006). Even though medical practitioners mostly name lack of time as the biggest obstacle for implementing spiritual care, previous research has shown that the spiritual competencies and personal spirituality of medical personnel best predict the implementation of these practices (Balboni et al., 2013; Bar-Sela et al., 2019; Best et al., 2015; Kristeller et al., 1999; Meredith et al., 2012; Olson et al., 2006).

In contrast to other medical skills, such as showing empathy or offering psychological interventions, SC has to do with the inner person of the recipient as well as of the caregiver. Thus, we presume that there is a connection between one´s own spirituality and one´s attitude and practice concerning SC. Awareness of and fostering one’s own spirituality has been associated with competencies in implementing appropriate and effective interventions in previous studies (Appleby et al., 2018; Assing-Hvidt et al., 2018; Baumann et al., 2011; Frick et al., 2019; Leeuwen, 2008; Marquardt & Demling, 2016). In a representative study in the USA with 1144 physicians, Franzen (2015) showed that their religious/spiritual orientation relates closely to religious and/or spiritual patient interactions: “The more central religion is for the physician, as reflected by their religious/spiritual orientation, intrinsic religiosity, and religious coping, the greater their perception of religion’s impact on health outcomes and their inclusion of religion/spirituality within clinical interactions” (Franzen, 2016, p. 1). Franzen in a later article also states that “‘religious and spiritual’ physicians include religion/spirituality most often” (Franzen, 2018, p. 1581). He also showed that training has an important direct effect on physicians’ actions. But he also showed that training is not associated with thinking that religion impacts patient health. “This means that training leads to greater inclusion but apparently not because of a shift in physicians’ thinking religion/spirituality impacts the health outcomes of their patients” (Franzen, 2016, p. 23).

The impact of physicians’ r/s orientation on their medical practice differs between cultures (Kørup et al., 2019). Recent literature also shows that the personal spirituality of health personnel is critical for SC: A study that included nine Middle Eastern countries (Bar-Sela et al., 2019) concluded that even across cultures, considering oneself not spiritual or only slightly spiritual was key for not providing SC. In fact, personal spirituality was the strongest factor that increased the number of cases in which SC was provided. In line with this, Curlin showed that physicians with heightened awareness of their own spirituality more often encourage patients to engage in spiritual conversations (Curlin et al., 2006; Rasinski et al., 2011) and enquire about spiritual issues (Curlin et al., 2006).

Worldwide research studies have shown that it is not only health professionals’ awareness but also their attitude towards spirituality that influences their interaction with patients. A meta-analysis concluded that physicians’ religiosity influences their clinical practice, thereby significantly altering patients’ health outcomes (Kørup et al., 2019). But not all findings point in this direction. A recent study has investigated SC practices among physicians in Brazil (Esperandio & Machado, 2019): Only a small percentage of the mainly Christian and highly religious physicians reported discussing spirituality with their patients.

Using his questionnaire on “Religion and Spirituality in Medicine: Physicians’ Perspectives” (Curlin et al., 2005), Curlin since 2002, and later other researchers, investigated not only whether physicians should inquire about patients’ religious and spiritual identity but also how doctors’ attitudes towards spirituality influence their interaction with patients (see the NERSH datapool, (Kørup et al., 2019)). In general, it seems that physicians who describe themselves being spiritual or religious are more likely to provide SC in different ways and acknowledge the rather positive effect spirituality can have on their patients (Curlin et al., 2005; Curlin et al., 2007a, 2007b; Kørup et al., 2019; Smyre et al., 2018). How proactively physicians should approach patients is discussed in literature. While some physicians are afraid to cross professional boundaries, others fear isolating patients from the SC they may need (Balboni, 2015; Balboni et al., 2011, 2013; Curlin et al., 2006; Scheurich, 2003; Smyre et al., 2018). According to Monroe et al. most primary care physicians do not consider patients' spirituality in routine medical care. However, if patients broach the subject themselves, most primary care physicians will give support (Monroe et al., 2003).

In this study, personal spirituality is defined as the individual’s approach to spiritual questions, regardless of whether they consider themselves religious or not.

As SC is a factor which affects the caregiver’s inner person, we hypothesised that in this field, there is an interrelationship between personal spirituality, spiritual competency and one’s attitude towards enquiring about spirituality in practice. This hypothesis is assessed in the present study which to our knowledge is the first one to test the hypothesis on German GPs.

## Research Questions

The research questions posed are:

1. Is there a correlation between GPs’ personal spirituality and their self-assessed spiritual competence?
2. Is there a correlation between GP’s personal spirituality and their attitude towards enquiring about spirituality in the practice?
3. Is there a correlation between GP´s self-assessed spiritual competence and their attitude towards enquiring about spirituality in practice?

## Method

### Study Design

In this cross-sectional study, correlation tests were performed using the data collected within the scope of the “Holistic Care Program for Elderly Patients to Integrate Spiritual Needs, Social Activity and Self-Care into Disease Management in Primary Care” (HoPES3) (Strassner et al., 2019).

For recruitment of practices, all family practices in the wider area of Heidelberg and Tübingen were contacted by mail. 30 GPs agreed to participate. As part of the HoPES3 baseline data assessment, 30 questionnaires filled in by GPs were evaluated. Amongst other topics, GPs were asked about their level of competence in providing SC, their own spirituality and attitude towards SC. The inclusion criterion for GPs was offering at least one disease management programme (DMP) for chronically ill patients. Disease Management Programs (DMPs) involve regular appointments and structured treatment plans designed to standardise and improve the quality of patient care in Germany. Inclusion criteria for patients were: age ≥ 70 years, suffering from ≥ 3 chronic conditions, taking ≥ 3 medications on a long-term basis and taking part in at least one DMP. Since this data set was part of a larger study, the necessary sample size was calculated using power criteria relevant for the primary objective of the study. For more details, see the study protocol (Strassner et al., 2019). For more results of the project see Mächler et al. (2022) and Sturm et al. (2022).

#### Measures

The analysed measures were collected as baseline data using the following survey instruments:

*Spiritual Care Competence* was measured using the Spiritual Care Competence Questionnaire (SCCQ) which is a scale quantifying self-assessed attributes of the SC competence of healthcare professionals. (Frick et al., 2019). The SCCQ has a seven-factor structure, comprising a total of 26 items. In a study validating the SCCQ, the seven factors accounted for 67% of the variance. The internal consistency of the overall construct is very good (*α* = 0.92). The seven factors are (1) *Perceptual competence* (the ability to perceive others’ spiritual and existential needs); (2) *Team-spirit* (the team’s actions in addressing spirituality); (3) *Documentation competence* (being sufficiently knowledgeable and able to assess and document patients’ spirituality and needs); (4) *Self-awareness and proactive opening competence* (specific actions intended both to deepen one’s own spirituality by partaking in events, seminars, etc. and to support patients’ spirituality by facilitating time and space for dialogue); (5) *Knowledge about other religions*; (6) *Interviewing and conversation competence* (the ability to conduct an open conversation about religious and existential topics); (7) *Proactive empowerment competence* (the ability to actively consider and foster patients’ spirituality).

In order to measure GPs’ awareness of their own spirituality, we used the following items:

Importance of spirituality for one’s own life; Personal spirituality as source for strength to overcome hardships; Importance of spirituality for one’s own health; “I know what I can draw strength and energy from,” “I regularly take time to make use of my sources of strength” were each measured with a 4-point Likert scale response option (0–3). The sum score (“Index”) of these items ranges from 0 to 15: the higher the value, the stronger the GPs’ awareness of their own spirituality. The items “Spirituality experienced as burdensome” (measured on a 4-point Likert scale as well, and “Affiliation to spiritual community” (measured as binary variable) were not included into the index calculation.

We further assessed GP’s attitude towards enquiring about spirituality in practice by asking whether they considered it to be a GP’s duty to enquire about faith or sources of strength in practice. These items were self-developed and built on experience from the literature (Monod et al., 2011). We had looked at different instruments in the literature for measuring spirituality and selected the items on this basis

Further, demographic data was collected (see Table 1).

**Table 1:** Sample description

Gender	18 male 12 female	
Family status	29 in partnership 1 not in partnership	
Religious denomination	None	8 (27%)
	Catholic	5 (16%)
	Protestant	17 (57%)
Years of professional experience	*N* =	30
	Mean	23 years
	Median	23 years
	Min. Max.	4.0–42.0 years
Personal spirituality as source for strength to overcome hardships	Not at all	2 (6.7%)
	A little bit	13 (43.3%)
	Considerably	7 (23.3%)
	A lot	8 (26.7%)
Importance of spirituality for one’s own life	Not at all	2 (6.7%)
	A little bit	12 (40.6%)
	Considerably	8 (26.7%)
	A lot	8 (26.7%)
Importance of Spirituality for one’s own health	Not at all	3 (10.3%)
	A little bit	10 (34.5%)
	Considerably	13 (44.8%)
	A lot	3 (10.3%)
I know what I can draw strength and energy from	Not at all	0 (–)
	Not quite true	1 (3.3%)
	Rather true	15 (50.0%)
	Exactly	14 (46.7%)
I regularly take time to make use of my sources of strength	Not at all	0 (–)
	Not quite true	9 (30.0%)
	Rather true	12 (40.6%)
	Exactly	9 (30.0%)
Personal spirituality (index)	*N* =	29
	Missing	1
	Mean	9.5
	SD	3.07
	Median	9
	Q1 Q3	7–12
	Min. Max.	5.0–15.0
Affiliation to spiritual community	No	15 (50.0%)
	Yes	15 (50.0%)
Spirituality experienced as burdensome	Never	11 (36.7%)
	Rarely	18 (60.0%)
	Often	1 (3.3%)
	Very often	0 (–)

### Statistical Methods

Descriptive statistical methods were used to analyse the data collected. Mean, standard deviation, median, first and third quartile, minimum and maximum were determined for continuous variables, while absolute and relative frequencies were calculated for ordinal and nominal variables. In order to assess the correlation between two variables with at least ordinal scale each, Spearman correlation coefficients were determined with 95% confidence intervals. If the absolute value of the correlation coefficient exceeded a value of 0.8, the correlation was considered very strong (denoted by ***), while absolute values between 0.5 and 0.8 suggested strong correlations (denoted by **), values between 0.2 and 0.5 indicated moderate or weak correlations (denoted by *), while coefficients with an absolute value between 0.2 and 0 were interpreted as a very weak correlation or none at all. The correlation between nominally scaled and ordinally scaled variables was assessed by conducting a (descriptive) two-sided Mann–Whitney–*U* test. The correlation between two nominally scaled variables was assessed via (descriptive) two-sided chi-squared tests. Resulting p-values are only to be interpreted descriptively, and thus, no adjustment for multiple testing was performed. *P*-values smaller than 0.05 were considered to be statistically significant (denoted by the symbol ^**X**^). The analysis was conducted using the statistical software R v3.6.4 and the package *DescTools*.

## Results

The sample description is shown in Table 1.

### Self-Assessed Spiritual Competence

We conducted analyses regarding the internal consistency of the SCCQ and the self-developed scale on spirituality. The Cronbach’s alpha for this sample’s SCCQ was calculated to be 0.90 with a 95% confidence interval of [0.84; 0.95], thus indicating good internal consistency for this scale. Cronbach’s alpha for the self-developed spirituality items was 0.76 with a 95% confidence interval of [0.64; 0.88], the point estimate indicating an acceptable internal consistency, even though the confidence interval is relatively broad, reflecting rather a large degree of uncertainty (Tables 2, 3 and 4).

**Table 2:** Associations between GPs’ personal spirituality and their self-assessed spiritual competence

Competence	Personal spirituality (index, showing how much influence spirituality has on one’s life)	Personal spirituality as source for strength to overcome hardships	Importance of spirituality for one’s own health	Spirituality experienced as wearing	Affiliation to spiritual community
	Spearman’s *ρ*				*p*-value (Mann–Whitney-*U*)
Documentation competence	0.080	0.232* 95%CI = [− 0.147; 0.551]	0.133	0,034	0.360
Competence in conversation technique	0.057	0.048	0.058	− 0,029	0.306
Knowledge about other religions	− 0.122	− 0.142	− 0.164	0,137	0.460
Team spirit	0.110	− 0.032	− 0.105	− 0,195	0.565
Perceptual competence	0.061	0.129	0.167	− 0,254	0.276
Proactive empowerment competence	0.296* 95%CI = [− 0.087; 0.603]	0.227* 95%CI = [− 0.152; 0.548]	0.248* 95%CI = [− 0.138; 0.568]	0,035	0.243
Self-awareness and proactive opening competence	0.828*** 95%CI = [0.659; 0.918]	0.848*** 95%CI = [0.695; 0.928]	0.733** 95%CI = [0.496; 0.869]	− 0,048	0.018^X^

**Table 3:** Associations between GPs’ personal spirituality and their attitude towards enquiring about spirituality in practice

Attitude	Personal spirituality (index, showing how much influence spirituality has on one’s life)	Personal spirituality as source for strength to overcome hardships	Importance of spirituality for one’s own health	Spirituality experienced as wearing	Affiliation to spiritual community
	Spearman’s *ρ*				*p*-value (Mann–Whitney-*U*)
Attitude towards enquiring about faith in practice	0.196	0.113	0.225* 95%CI = [− 0.161; 0.552]	− 0,031	0.488
Attitude towards enquiring about sources of strength in practice	0.385* 95%CI = [− 0.022; 0.659]	0.359* 95%CI = [− 0.001; 0.637]	0.359* 95%CI = [0.012; 0.639]	− 0,198	0.212

**Table 4:** Correlations between GPs’ self-assessed spiritual competence and their attitude towards enquiring about spirituality in practice

	Attitude towards enquiring about sources of strength in the practice	Attitude towards enquiring about faith in the practice
Competence	Spearman’s *ρ*	
Documentation competence	0.011	− 0.178
Competence in conversation technique	0.471* 95%CI = [0.133; 0.711]	0.171
Knowledge about other religions	0.315* 95%CI = [− 0.051; 0.606]	0.147
Team spirit	0.153	− 0.113
Perceptual competence	0.669** 95%CI = [0.401; 0.832]	0.229* 95%CI = [− 0.158; 0.554]
Proactive empowerment competence	0.457* 95%CI = [0.109; 0.706]	0.222* 95%CI = [− 0.165; 0.549]
Self-awareness and proactive opening competence	0.505** 95%CI = [0.162; 0.739]	0.150

We found correlations between GPs’ *Self-awareness and proactive opening competence* and personal spirituality as a source for strength (*ρ* = 0,848) as well as for health (*ρ* = 0,733). *Self-awareness and proactive opening competence* further strongly correlated with GPs’ personal spirituality and its general influence on their lives (*ρ* = 0,828).

Weak correlation was found between GPs’ personal spirituality (*ρ* = 0,296), their personal spirituality as a source for strength (*ρ* = 0,227) as well as for health (*ρ* = 0,248) and their *Proactive empowerment competence.*

Also, weak correlation was found between GPs’ *Documentation competence* (*ρ* = 0,232) and their personal spirituality as a source for strength.

*Conversation technique, Knowledge about other religions, Team spirit and Perceptual competence* did not show significant correlation with GPs’ personal spirituality.

Moderate correlations were found between GPs’ attitude towards enquiring about sources of strength in practice and their personal spirituality as a source for strength to overcome hardships (*ρ* = 0.359), their personal spirituality regarding their own health (*ρ* = 0.359), as well as their personal spirituality (Index) (*ρ* = 0.385). The attitude of GPs towards enquiring about faith was less correlated with personal spirituality than their attitude towards enquiring about sources of strength, which means that enquiring about faith is stronger connected to the personal spirituality of the GP than enquiring about sources of strength.

GPs’ *Perceptual competence* strongly correlated (*ρ* = 0,669) with their attitude towards enquiring about *sources of strength* in practice. The latter correlated weakly or moderately with a number of other factors, namely that of their *Conversational competence* (*ρ* = 0,471), their *Knowledge about other religions* (ρ = 0,315), their *Proactive empowerment competence* (ρ = 0,457) and their *Self-awareness and proactive opening competence* (ρ = 0.505).

In addition, enquiring about *faith* in practice moderately *correlated* with GPs’ *Perceptual competence* (*ρ* = 0,229) as well as their *Proactive empowerment competence* (*ρ* = 0,222).

Generally, more GPs considered enquiring about sources of strength to be appropriate compared to asking patients about faith.

## Discussion

### Summary

This analysis of baseline data looking at 30 GPs shows some connections between different SC aspects:

- Many GPs acknowledge that spiritual care should be an integral part of primary care
- However, previous research shows a lack of education and competencies to fulfil this task
- Spiritual competencies include discerning, addressing and meeting patients’ spiritual needs
- Personal spiritual resources (and not necessarily a religious affiliation) are associated with GPs’ spiritual competence.

In the following we discuss GPs’ personal spirituality, spiritual competence and attitude towards SC as relevant factors for the implementation of SC in general practice.

### Strengths and Limitations

While addressing German GPs’ personal spirituality and spiritual competence for the first time, which is a strength of the study, it should be noted that we asked GPs to assess their own individual competence in providing SC. Their patients were not asked for their opinions. Patients might have a very different view on their doctors’ competence in this field (Baumann et al., 2011; Best et al., 2016; Marquardt & Demling, 2016). We have to assume that there is a selection bias in favour of those GPs who are already interested in SC. Hence, this aspect should be taken into account when interpreting the results presented above. The presented cross-sectional study only included a relatively small number of GPs (*N* = 30), which limits the representativity of the sample and the generalizability of the results found. This also leads to uncertainty with regard to the correlations performed, which is reflected in the resulting broad confidence intervals, and to a low statistical power of the descriptively used statistical tests.

### Discussion Against the Background of the Existing Literature

#### Personal Spirituality and Spiritual Competence

A correlation was found between GPs’ personal spirituality and their self-assessed spiritual competence.

Our results indicate that the findings of studies in other countries are also true for German GPs. They also confirm what literature indicates about the connection between one’s personality, one’s own spirituality and one’s attitude and practice concerning SC. In SC, “being” is as relevant as “doing” and personal awareness of one’s own spirituality is essential in order to meet patients’ spiritual needs (Baldacchino, 2015).

Our findings suggest that training which helps participants to reflect on their own spirituality might be more effective in developing spiritual competence than training that focuses on imparting knowledge. According to Bar-Sela (Bar-Sela et al., 2019), training programmes designed to expand and improve SC provision by healthcare staff include elements aiming to engage participants and address their own spirituality. Research on the effect of these interventions show a measurable increase in SC provision. In line with this, Baldacchino (Baldacchino, 2015) states: “If you reform your spiritual-self, you will reform your professional care.”

#### Personal Spirituality and Attitude Towards SC

The data shows moderate correlation between GPs’ attitude towards enquiring about *sources of strength* in practice and their personal spirituality. The attitude towards enquiring about *faith* was less correlated with personal spirituality.

The vast majority of GPs (89%) included in our study considered it appropriate to enquire about their patents’ sources of strength. This gives the impression that GPs are generally open to taking on this task. One of the GPs in the study stated: “We are the ones who accompany in extreme circumstances, in disease and death. Who if not us?”.

The results of our study support the assumption that GPs’ personal spirituality has an influence on their attitude towards SC in practice, thereby adding novel data on German GPs to the literature.

In summary, these results suggest that training programmes targeting GPs’ personal spirituality and enabling reflection on their own spirituality could be effective in shaping GPs ‘ attitude towards the integration of SC in practice, and thus foster the provision of SC.

#### Spiritual Competence and Attitude Towards SC

We found several weak to moderate correlations between GPs’ attitude to enquiring about sources of strength or faith in practice on the one hand, and on the other hand their self-assessed spiritual competencies, namely *Self-awareness and proactive opening competence* and *Perceptual competence*. Concerning *Self-awareness and proactive opening competence* we would have expected a stronger correlation. However, there was a strong correlation between GPs´ *Perceptual competence* and the aforementioned attitude.

What could this mean for activities aiming to promote the provision of SC?

The results indicate a positive correlation between the ability to perceive the spiritual and existential needs of others, and a sense of duty to attend to these needs. We can assume that if doctors are not able to discern patients’ wishes to talk about their spiritual needs, GPs will miss the opportunity to provide the space for conversation and to offer support. Insufficient training offered at medical school as well as insufficient ongoing training through medical education programmes seems to be a substantial part of the problem (Balboni et al., 2013; Epstein-Peterson et al., 2015; Selman et al., 2018). Taverna et al (2019) report that in a survey conducted in three German-speaking countries most of the teaching on SC was organised in classes on a voluntary basis. Mandatory courses aiming to improve the perceptual competency of the profession in the field of SC might offer a solution.

## Conclusion

It would be desirable to conduct a follow-up study to re-assess our findings with a larger sample size, but there is no funding for it yet. Given the innovative character of the study, being the first to focus on German GPs and their spirituality, we consider the results nevertheless valuable. However, one should not rely on the point estimates alone in the interpretation.

The present study suggests interrelation between German GPs' personal spirituality, their spiritual competence and their readiness to accept SC as their duty. The results support the conclusion that if professional training and evaluation in General Practice aim to strengthen SC, spiritual competence and reflection on personal spirituality should be addressed at all stages of medical education as well as in quality management.

## References

[CR1] Appleby A, Swinton J, Bradbury I, Wilson P (2018). GPs and spiritual care: Signed up or souled out? A quantitative analysis of GP trainers' understanding and application of the concept of spirituality. Education for Primary Care.

[CR2] Appleby A, Swinton J, Wilson P (2019). Spiritual care training and the GP curriculum: Where to now?. Education for Primary Care.

[CR3] Assing-Hvidt E, Ammentorp J, Søndergaard J, Timmermann C, Hansen DG, Hvidt NC (2018). Developing and evaluating a course programme to enhance existential communication with cancer patients in general practice. Scandinavian Journal of Primary Health Care.

[CR4] Assing Hvidt E, Sondergaard J, Ammentorp J, Bjerrum L, Gilsa Hansen D, Olesen F, Pedersen SS, Timm H, Timmermann C, Hvidt NC (2016). The existential dimension in general practice: Identifying understandings and experiences of general practitioners in Denmark. Scandinavian Journal of Primary Health Care.

[CR5] Balboni MJ (2015). Everyday religion in hospitals. Society.

[CR6] Balboni MJ, Babar A, Dillinger J, Phelps AC, George E, Block SD, Kachnic L, Hunt J, Peteet J, Prigerson HG, VanderWeele TJ, Balboni TA (2011). "It depends": Viewpoints of patients, physicians, and nurses on patient-practitioner prayer in the setting of advanced cancer [research support, non-U.S. Gov't]. Journal of Pain and Symptom Management.

[CR7] Balboni, M. J., Sullivan, A., Amobi, A., Phelps, A. C., Gorman, D. P., Zollfrank, A., Peteet, J. R., Prigerson, H. G., VanderWeele, T. J., & Balboni, T. A. (2013). Why is spiritual care infrequent at the end of life? Spiritual care perceptions among patients, nurses, and physicians and the role of training. *Journal of Clinical Oncology,**31*(4), 461–467. https://ascopubs.org/doi/10.1200/JCO.2012.44.6443 10.1200/JCO.2012.44.6443 PMC4878036 23248245

[CR8] Balboni TA, Vanderwerker LC, Block SD, Paulk ME, Lathan CS, Peteet JR, Prigerson HG (2007). Religiousness and spiritual support among advanced cancer patients and associations with end-of-life treatment preferences and quality of life. Journal of Clinical Oncology.

[CR9] Baldacchino D (2015). Spiritual Care education of health care professionals. Religions.

[CR10] Bar-Sela G, Schultz MJ, Elshamy K, Rassouli M, Ben-Arye E, Doumit M, Gafer N, Albashayreh A, Ghrayeb I, Turker I (2019). Training for awareness of one's own spirituality: A key factor in overcoming barriers to the provision of spiritual care to advanced cancer patients by doctors and nurses. Palliative & Supportive Care.

[CR11] Baumann K, Lee E, Zahn A (2011). ‘Religion in psychiatry and psychotherapy?’ A pilot study: The meaning of religiosity/spirituality from staff’s perspective in psychiatry and psychotherapy. Religions.

[CR12] Best M, Butow P, Olver I (2015). Do patients want doctors to talk about spirituality? A systematic literature review. Patient Education and Counseling.

[CR13] Best M, Butow P, Olver I (2016). Doctors discussing religion and spirituality: A systematic literature review. Palliative Medicine.

[CR14] Büssing A, Michalsen A, Balzat H-J, Grünther R-A, Ostermann T, Neugebauer EAM, Matthiessen PF (2009). Are spirituality and religiosity resources for patients with chronic pain conditions?. Pain Medicine.

[CR15] Curlin FA, Chin MH, Sellergren SA, Roach CJ, Lantos JD (2006). The association of physicians' religious characteristics with their attitudes and self-reported behaviors regarding religion and spirituality in the clinical encounter. Medical Care.

[CR16] Curlin FA, Lantos JD, Roach CJ, Sellergren SA, Chin MH (2005). Religious characteristics of U.S. physicians: A national survey. Journal of General Internal Medicine.

[CR17] Curlin FA, Lawrence RE, Chin MH, Lantos JD (2007). Religion, conscience, and controversial clinical practices. New England Journal of Medicine.

[CR18] Curlin FA, Sellergren SA, Lantos JD, Chin MH (2007). Physicians' observations and interpretations of the influence of religion and spirituality on health. Archives of Internal Medicine.

[CR19] Ellis MR, Vinsor D, Ewigman B (1999). Addressing spiritual concerns of patients: Family physicians' attitudes and practices. Journal of Family Practice.

[CR20] Epstein-Peterson ZD, Sullivan AJ, Enzinger AC, Trevino KM, Zollfrank AA, Balboni MJ, VanderWeele TJ, Balboni TA (2015). Examining forms of spiritual care provided in the advanced cancer setting. American Journal of Hospice and Palliative Care.

[CR21] Esperandio MRG, Machado GAS (2019). Brazilian physicians’ beliefs and attitudes toward patients’ spirituality: Implications for clinical practice. Journal of Religion & Health.

[CR22] Franzen AB (2015). Physicians in the USA: Attendance, beliefs and patient interactions. Journal of Religion and Health.

[CR23] Franzen AB (2016). Is this relevant? Physician perceptions, clinical relevance, and religious content in clinical interactions. Journal for the Scientific Study of Religion.

[CR24] Franzen AB (2018). Influence of physicians’ beliefs on propensity to include religion/spirituality in patient interactions. Journal of Religion and Health.

[CR25] Frick E, Riedner C, Fegg MJ, Hauf S, Borasio GD (2006). A clinical interview assessing cancer patients' spiritual needs and preferences. European Journal of Cancer Care.

[CR26] Frick E, Theiss M, Rodrigues Recchia D, Büssing A (2019). Validierung einer deutschsprachigen Skala zur Messung der spiritual care Kompetenz. Spiritual Care.

[CR27] Hodge DR (2007). The spiritual competence scale: A new instrument for assessing spiritual competence at the programmatic level. Research on Social Work Practice.

[CR28] Kørup AK, Søndergaard J, Lucchetti G, Ramakrishnan P, Baumann K, Lee E, Frick E, Büssing A, Alyousefi NA, Karimah A (2019). Religious values of physicians affect their clinical practice: A meta-analysis of individual participant data from 7 countries. Medicine.

[CR29] Kristeller JL, Sheedy Zumbrun C, Schilling RF (1999). "I would if I could": How oncologists and oncology nurses address spiritual distress in cancer patients. Psycho-Oncology.

[CR30] Leeuwen RR (2008). Towards nursing competencies in spiritual care.

[CR42] Mächler R, Sturm N, Frick E, Schalhorn F, Stolz R, Valentini J, Krisam J, Straßner C (2022). Evaluation of a spiritual history with elderly multi-morbid patients in general practice—a mixed-methods study within the project HoPES3. International Journal of Environmental Research & Public Health.

[CR31] Marquardt M, Demling JH (2016). Psychotherapie und religion: Eine repräsentative Umfrage unter Psychotherapeuten in Süddeutschland. Psychotherapie Psychosomatik Medizinische Psychologie.

[CR32] Meredith P, Murray J, Wilson T, Mitchell G, Hutch R (2012). Can spirituality be taught to health care professionals?. Journal of Religion & Health.

[CR33] Monod S, Brennan M, Rochat E, Martin E, Rochat S, Büla CJ (2011). Instruments measuring spirituality in clinical research: A systematic review. Journal of General Internal Medicine.

[CR34] Monroe MH, Bynum D, Susi B, Phifer N, Schultz L, Franco M, MacLean CD, Cykert S, Garrett J (2003). Primary care physician preferences regarding spiritual behavior in medical practice. Archives of Internal Medicine.

[CR35] Olson M, Sandor MK, Sierpina V, Vanderpool H, Dayao P (2006). Mind, body, and spirit: Family physicians’ beliefs, attitudes, and practices regarding the integration of patient spirituality into medical care. Journal of Religion & Health.

[CR36] Rasinski KA, Kalad YG, Yoon JD, Curlin FA (2011). An assessment of US physicians’ training in religion, spirituality, and medicine. Medical Teacher.

[CR37] Scheurich N (2003). Reconsidering spirituality and medicine. Academic Medicine.

[CR38] Selman LE, Brighton LJ, Sinclair S, Karvinen I, Egan R, Speck P, Powell RA, Deskur-Smielecka E, Glajchen M, Adler S, Puchalski CM, Hunter J, Gikaara N, Hope J (2018). Patients’ and caregivers’ needs, experiences, preferences and research priorities in spiritual care: A focus group study across nine countries. Palliative Medicine.

[CR39] Smyre CL, Tak HJ, Dang AP, Curlin FA, Yoon JD (2018). Physicians' opinions on engaging patients' religious and spiritual concerns: A national survey. Journal of Pain and Symptom Management.

[CR40] Strassner C, Frick E, Stotz-Ingenlath G, Buhlinger-Göpfarth N, Szecsenyi J, Krisam J, Schalhorn F, Valentini J, Stolz R, Joos S (2019). Holistic care program for elderly patients to integrate spiritual needs, social activity, and self-care into disease management in primary care (HoPES3): Study protocol for a cluster-randomized trial. Trials.

[CR43] Sturm N, Krisam J, Szecsenyi J, Bentner M, Frick E, Mächler R, Schalhorn F, Stolz R, Valentini J, Joos S, Straßner C (2022). Spirituality, self-care, and social activity in the primary medical care of elderly patients—results of a cluster-randomized interventional trial (HoPES3). Deutsches Ärzteblatt international.

[CR41] Taverna M, Sattel H, Berberat P, Frick E (2019). A survey on the integration of spiritual care in medical schools from the German speaking faculties. Advances in Medical Education and Practice.

